# Quantifying the HDL proteome by mass spectrometry: how many proteins truly associate with HDL?

**DOI:** 10.1002/ejhf.1166

**Published:** 2018-03-02

**Authors:** Gunther Marsche

**Affiliations:** ^1^ Otto Loewi Research Center, Division of Pharmacology Medical University of Graz Austria

I read with great interest the article by Emmens and colleagues published in the *European Journal of Heart Failure*.^1^ The authors purposed to examine whether changes in the high‐density lipoprotein (HDL) proteome of heart failure patients relate to a worse prognosis. Certainly, Emmens *et al.* have conducted a very interesting and valuable study, but some methodological issues need to be considered. Because of differences in HDL isolation techniques and poor purification of the isolated HDL fractions, the total ‘list’ of HDL proteins unfortunately varies dramatically from study to study, and it is not clear what proteins truly associate with HDL. When studying the HDL proteome, it requires highly purified fractions of HDL, using a two‐step protocol, consisting of density gradient ultracentrifugation (d = 1.063–1.21 g/mL) or immunoprecipitation, followed by size exclusion chromatography to minimize contamination with low density lipoproteins and other lipidated plasma proteins that are co‐isolated with HDL.^2^, ^3^ Emmens *et al.* used a calcium silicate matrix to separate lipoproteins from other plasma proteins, which is not sufficient to yield highly purified fractions of HDL. In addition, no quantitative raw data were provided, making it impossible for the reader to judge the quality of lipoprotein preparations and the abundance of the individual proteins. Therefore, it is not clear whether the identified proteins are HDL‐associated.

## Funding

This work was supported by the Austrian Science Fund FWF (Grants P22976‐B18 and W1241).
